# Loneliness in Schizophrenia: The Mediating Roles of Asocial Beliefs and Mattering

**DOI:** 10.1177/00207640251382463

**Published:** 2025-10-31

**Authors:** Miya M. Gentry, Molly A. Patapoff, Sophia Ross, Allison P. Williams, Barton W. Palmer

**Affiliations:** 1 San Diego State University/University of California San Diego Joint Doctoral Program in Clinical Psychology, USA; 2 Department of Psychiatry, University of California San Diego, La Jolla, USA; 3 Veterans Affairs San Diego Healthcare System, VISN 22 Mental Illness Research, Education, and Clinical Center (MIRECC), CA, USA

**Keywords:** social connection, isolation, psychosis, social cognition, social functioning

## Abstract

**Background:**

Loneliness is a pervasive concern for people with schizophrenia and is associated with deleterious effects on health outcomes. However, its underlying mechanisms remain insufficiently understood.

**Aims:**

The current study examined the roles of perceptions of mattering to others and asocial beliefs in contributing to loneliness among people with schizophrenia compared to those without a history of serious mental illness (NC).

**Methods:**

Participants included people with schizophrenia (*N* = 72) and an NC group (*N* = 65), aged 41 to 70 years, from a parent study of loneliness and aging. Group differences in loneliness, asocial beliefs, and mattering were assessed. Path analyses tested a partial mediation model evaluating direct and indirect pathways between diagnostic group, asocial beliefs, mattering, and loneliness.

**Results:**

People with schizophrenia reported higher loneliness and asocial beliefs and lower perceived mattering than the NC group. Path analyses indicated that asocial beliefs and mattering partially mediated the relationship between diagnostic group and loneliness. Specifically, higher asocial beliefs were associated with lower mattering, which in turn predicted greater loneliness.

**Conclusions:**

Our findings highlight the potential value of evaluating and addressing asocial beliefs and perceptions of mattering as novel targets in intervention to reduce loneliness among people with schizophrenia.

## Introduction

Persistent loneliness is a significant concern for many people with schizophrenia (Badcock et al., 2015; Beebe, 2010; Stain et al., 2012). Chronic loneliness has far-reaching consequences, negatively affecting mental and physical health, overall well-being, and even mortality (Murthy, 2023). For people with schizophrenia, loneliness may be compounded by social withdrawal, stigma, and cognitive impairments, which collectively reinforce social isolation and exacerbate negative outcomes (Michalska da Rocha et al., 2018). Experiences of mental health stigma and internalized negative beliefs (e.g. diminished self-worth, self-blame, or defeatist attitudes) restrict opportunities for social participation and can lead to diminished social networks, which in turn worsen functional outcomes and quality of life (Cavieres et al., 2023; Fulford & Holt, 2023). Such processes may undermine a person’s sense of social value and reinforce maladaptive beliefs about relationships, setting the stage for loneliness. Therefore, understanding the mechanisms driving loneliness in this population is critical for developing effective interventions that enhance social connectedness and reduce isolation, interrelated goals that promote both better psychological and physical well-being. One potential protective factor against loneliness is perceived mattering, defined as the belief that one is valued and important to others (Flett, 2022).

A sense of mattering to others has been consistently linked to enhanced emotional well-being in the general population (Bowman et al., 2022; McComb et al., 2020; Paradisi et al., 2024). Prior research regarding loneliness, primarily in non-clinical samples, has demonstrated a strong negative correlation between perceived mattering and loneliness (Flett et al., 2016; McComb et al., 2020; Shafiq et al., 2024). Importantly, this association is not tautological; mattering is neither the mere inverse of loneliness nor synonymous with the ‘need to belong’ (Flett, 2022). Whereas loneliness reflects subjective distress arising from a perceived discrepancy in the quantity or quality of one’s social relationships (Cacioppo & Patrick, 2008), mattering pertains to the perceived significance of oneself to others (Flett, 2022).

To our knowledge, no quantitative studies have directly examined the association between mattering and loneliness among people with schizophrenia. However, related research in serious mental illness (SMI) populations has shown that mattering mediates the relationship between social support, recovery, and internalized stigma (Pernice et al., 2017). This construct is highly relevant to schizophrenia, where individuals often encounter stigma, social rejection, and internalized negative beliefs that may erode their sense of social value (Bowman et al., 2022; Neumann et al., 2023). Findings from Pernice et al. (2017) suggest that, while stigma is a broad construct, one of its key consequences may be a diminished sense of mattering. Such diminished mattering may, in turn, increase vulnerability to loneliness among people with schizophrenia. Reinforcing mattering could therefore represent a promising avenue for mitigating loneliness by affirming the importance of individuals’ social presence and participation.

The concept of mattering contrasts with another key variable frequently observed in schizophrenia – asocial beliefs. Asocial beliefs include pessimistic or maladaptive views of social interactions, including perceptions that social engagement is unpleasant, burdensome, or unlikely to be rewarding (Granholm et al., 2018; Grant & Beck, 2010). Asocial beliefs may manifest as aversive social beliefs or behaviors (e.g. actively avoiding social interactions due to anticipated discomfort), social distancing beliefs (e.g. preference for isolation), and a devaluation of social relationships (e.g. viewing friendships as unimportant and not worth the effort). While devaluation may, in some cases, protect against the subjective distress of loneliness, it can still undermine social functioning and perpetuate disengagement via cognitive distortions about those interactions and the perceptions others have of oneself (Granholm et al., 2018; Grant & Beck, 2010). Regarding the association of asocial beliefs and mattering, the presence of social beliefs may erode perceptions of mattering, reinforcing the cycle of loneliness and social disengagement (Granholm et al., 2018; Grant & Beck, 2010). However, to our knowledge, there have been no prior studies of the three-way associations among mattering, asocial beliefs, and loneliness specifically in people with schizophrenia.

These factors are central to understanding social functioning, defined as the capacity to engage in and sustain meaningful interpersonal roles (Bosc, 2000). Social functioning deficits are among the strongest predictors of poor outcomes in schizophrenia (e.g. greater symptom severity and reduced quality of life), with meta-analyses showing broad impairments in social skills and relationships (Michalska da Rocha et al., 2018). Within this broader context, asocial beliefs may exacerbate loneliness by reinforcing social withdrawal (Granholm et al., 2018; Grant & Beck, 2010), whereas mattering may buffer against decline by strengthening one’s sense of social value (McComb et al., 2020).

To determine whether these associations are specific to schizophrenia or reflect broader patterns in the general adult population, the present study included a Non-SMI (NC) comparison group. Therefore, the current study was designed to examine whether asocial beliefs and mattering mediate the relationship between diagnostic group and loneliness. Based on the above considerations, we hypothesized that, compared to the NC group, people with schizophrenia would report higher asocial beliefs and loneliness but lower perceived mattering. We further hypothesized that diagnostic group would predict loneliness, both directly and indirectly through pathways of asocial beliefs and mattering, with higher asocial beliefs associated with lower perceptions of mattering, which in turn would contribute to explaining group differences in loneliness. If supported, these findings point to novel intervention targets, namely challenging asocial beliefs and enhancing perceptions of mattering to improve social functioning and reduce loneliness in schizophrenia.

## Methods

### Participants

Data for the current study were collected as part of a recently completed parent study of loneliness and aging in schizophrenia. Participants for the current analyses included 137 adults, including 72 (52.6%) participants with schizophrenia or schizoaffective disorder and 65 (47.4%) participants without a history of SMI (NC) group. Due to the design of the parent study, participant enrollment was restricted to persons between ages 41 and 70 years. The current study was approved by the UC San Diego Office of IRB Administration and conducted in accordance with the ethical principles of the Declaration of Helsinki.

People with schizophrenia were recruited through presentations and printed flyers at county area community-based residential Board-and-Care facilities, clubhouse programs in the greater San Diego area, and a registry of participants in prior studies who consented to contact for future research participation. Recruitment for NC participants was done through community flyers, ResearchMatch.com, as well as the above-mentioned registry of people indicating potential interest in future research participation. Data for the present analyses were collected between March 2021 and November 2024.

In addition to the above-listed age criterion, inclusion in the parent study required a clinical diagnosis of schizophrenia or schizoaffective disorder, established by the clinical provider or via review of prior records. For the NC group, an absence of a history of SMI was determined through interview reviewing history of relevant DSM-5 symptoms of psychosis or bipolar disorder, or a history of depression that was sufficiently severe to require hospitalization. Other inclusion criteria were English fluency and the provision of informed written consent using an IRB-approved consent form. Exclusion criteria included plans to move outside San Diego within 12 months, dementia or neurological conditions affecting cognition, active substance use disorders, and medical conditions impairing study participation. In addition, for inclusion in the present analyses, we required complete data on the UCLA-Loneliness Scale, General Mattering Scale, and Asocial Beliefs Questionnaire described in detail below. Of 157 otherwise eligible participants, 20 (5 with schizophrenia and 15 NCs) were excluded due to incomplete data. Although the parent study includes 6- and 12-month follow-up visits, the present analyses are focused on the interrelationship among the baseline measures.

### Measures

#### Sociodemographic Characteristics

Information on age, gender, and race/ethnicity characteristics was gathered through self-report during interviews and/or self-administered rating scales.

#### Loneliness

Loneliness was measured using the 20-item UCLA Loneliness Scale – Third Edition (UCLA-LS; Russell, 1996). The UCLA-LS total scores have a potential range from 20 to 80 points, with higher scores reflecting worse loneliness. It has been the most widely used measure in loneliness research, and has excellent internal consistency (Cronbach’s α > .88) and test-retest reliability (*r* = .73; Russell, 1996).

#### Asocial Beliefs

Asocial beliefs were assessed using the 15-item Asocial Beliefs Scale (ABS; Grant & Beck, 2010). The ABS measures maladaptive cognitions about social interactions, including defeatist, avoidant, and pessimistic attitudes toward forming and maintaining relationships. These beliefs reflect distorted expectations that social engagement is unpleasant, burdensome, or unrewarding. Sample items include, “People are usually better off if they stay aloof from emotional involvements with most others,” and “People usually expect too much of others.” Responses are rated “true” or “false,” with higher scores (range = 0–15) indicating more severe asocial beliefs. This scale has shown good internal consistency (Cronbach’s α = .76) and test-retest reliability (*r* = .85; Grant & Beck, 2010)

#### Mattering

Mattering was measured by the General Mattering Scale (GMS; Marcus & Rosenberg, 1987). It is a unidimensional measure consisting of five items that assess an individual’s self-perceived significance to others. Items are rated on a 4-point scale ranging from 1 (Not at all) to 4 (A lot). The GMS is a widely used measure of mattering, assessing perceptions of one’s significance to others (Flett, 2018). Example items include “How important do you feel you are to other people?” and “How interested are people generally in terms of what you have to say?” The GMS has shown good internal consistency (Cronbach’s α = .78; Taylor & Turner, 2001a) and test-retest reliability (*r* = .83; Mahamid et al., 2024).

### Statistical Analyses

All analyses were conducted in R (R Core Team, 2020). Descriptive statistics were computed using the *dplyr* package, and multivariate analyses were performed with the *lavaan* package. Non-normality was assessed using both visual inspection of histograms and the Shapiro-Wilk test. Results indicated skewness and moderate departures in kurtosis across the three primary variables (i.e. loneliness, asocial beliefs, and mattering). Given these violations of normality, group differences in mean scores were evaluated using the non-parametric Wilcoxon Sign-Rank Test. A path analysis specifying a partial mediation model was conducted to examine the relationships between diagnostic group (NC subjects coded as 0; people with schizophrenia coded as 1), asocial beliefs, mattering, and loneliness. To account for the observed skewness and kurtosis amongst continuous variables, a robust maximum likelihood estimation (MLR) was employed to generate standard errors and confidence intervals.

The partial mediation model assessed whether asocial beliefs and mattering mediated the relationship between diagnostic group and loneliness while retaining the direct effect of the diagnostic group on loneliness. Given empirical literature suggesting that loneliness can differ based on these factors, covariates of age, gender, and race/ethnicity were included in the model (Dahlberg et al., 2018). Model fit was evaluated using multiple scaled-fit indices: the chi-square statistic ( $\chi^{2}$ ), Comparative Fit Index (CFI), Tucker-Lewis Index (TLI), Root Mean Square Error of Approximation (RMSEA) with 90% confidence intervals, and the Standardized Root Mean Square Residual (SRMR). Model fit criteria included a CFI ⩾0.95, TLI ⩾0.95, RMSEA ⩽0.08, and SRMR ⩽0.05 for goodness of fit (Bentler, 1990; Hu & Bentler, 1999; Steiger, 1990). The model results included standardized and unstandardized path coefficients, and statistical significance was determined at *p* < .05. All effect sizes were determined by Cohen’s (1988) guidelines.

## Results

### Sample Characteristics

The sample sociodemographic characteristics and descriptive statistics are summarized in Table 1. Significant group differences were found for loneliness (*W* = 1,242, *p* < .001, *r* = .476, moderate effect), asocial beliefs (*W* = 1,220.5, *p* < .001, *r* = .458, moderate effect), and mattering (*W* = 3,497.5, *p* = .002, *r* = .257, small effect). Specifically, relative to the NC group, the schizophrenia group had significantly higher loneliness and asocial beliefs scores. On average, people with schizophrenia UCLA-LS total scores were 8.7 points higher than those in the NC group.

**Table 1.** Sociodemographic Characteristics.

	Schizophrenia (*n* = 72)	NC (*n* = 65)			
*Sample Characteristics*	*M* (*SD*) or *n* (%)	M (*SD*) or *n* (%)	*t* or $\chi^{2}$	df	*p*
Age	55.0 (7.5)	57.4 (*8.6)*	1.95	148.19	.053
Gender			9.73	1	.002
Women (%)	31 (37.8%)	48 (64.0%)			
Men (%)	51 (62.2%)	27 (36.0%)			
Race/ethnicity			22.30	6	.001
White	39 (47.6%)	54 (72.0%)			
African-American/Black	15 (18.3%)	2 (2.7%)			
Hispanic/Latine	18 (22.0%)	10 (13.3%)			
Asian	4 (4.9%)	9 (12.0%)			
Other	6 (7.3%)	-			
*Key variables*					
Loneliness (UCLA-LS total)	46.8 *(10.7)*	35.6 *(9.93)*			
Asocial beliefs (ABS total)	5.73 *(3.24)*	2.97 *(2.88)*			
Mattering (GMS total)	14.3 *(4.43)*	16.7 *(3.01)*			

*Note.* NC = Non-SMI comparison group. Group differences in categorical variables (e.g. gender and race/ethnicity) were assessed using chi-square tests; Differences in continuous variables (e.g. age) were assessed using an independent samples *t*-test. Reported *t* or $\chi^{2}$ values reflect test statistics comparing schizophrenia and NC group. “Hispanic/Latine” refers to participants who self-identified as having Hispanic or Latine ethnic backgrounds. More detailed subcategories of race/ethnicity were not collected. UCLA-LS = UCLA Loneliness Scale – Third Edition; ASB = Asocial Beliefs, GMS = General Mattering Scale).

### Direct and Indirect Effects of Diagnostic Group on Loneliness

**Figure 1.**
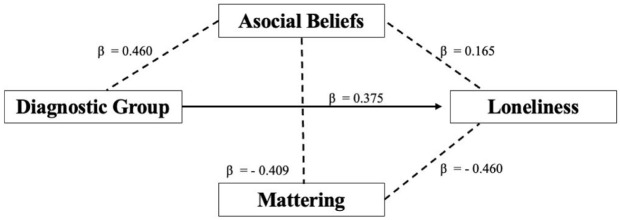
Direct and indirect pathways to loneliness.

A partial mediation model using the MLR estimator examined the relationships among diagnostic group, asocial beliefs, mattering, and loneliness, with asocial beliefs and mattering specified as mediators of the relationship between diagnostic group and loneliness (see Figure 1). Model fit indices indicated acceptable fit ( [1] = 3.009, *p* = .056), with a high Comparative Fit Index (CFI = 0.986) and low SRMR (0.025). However, the RMSEA (0.126) and TLI (0.784) suggest room for model improvement. The model explained 51.8% of the variance in loneliness (*R*^2^ = .518), 20.5% of the variance in asocial beliefs (*R*^2^ = .205), and 18.6% of the variance in mattering (*R*^2^ = .186).

Consistent with the group comparison analyses above, the direct effect of the diagnostic group on loneliness was statistically significant (*B* = 8.726, β = .375, *p* < .001). The indirect effect of diagnostic group on loneliness through asocial beliefs and mattering was also statistically significant (*B* = 2.009, β = .086, *p* = .002, 95% CI [0.733, 3.286]). Although small, this effect indicates that changes in asocial beliefs and mattering are associated with loneliness in people with schizophrenia. The total effect of diagnostic group on loneliness was statistically significant (*B* = 10.735, β = .461, *p* < .001, 95% CI [7.802, 13.668]), indicating that both direct and indirect pathways contributed to the overall influence of diagnostic group on loneliness.

All path coefficients and respective test statistics can be found in Table 2. Further analysis revealed that diagnostic group significantly predicted asocial beliefs, indicating that people with schizophrenia reported higher levels of asocial beliefs compared to the NC group. This relationship represents a moderate effect, suggesting a notable difference between the two groups. In turn, higher levels of asocial beliefs significantly predicted lower perceptions of mattering, meaning that those who endorsed more asocial beliefs also felt less like they mattered to others, reflecting a moderate effect. Perceptions of mattering significantly predicted loneliness, suggesting that individuals who felt less important or valued tended to experience greater loneliness. This relationship suggested a strong effect, emphasizing the crucial role of feeling like one matters in mitigating loneliness. Moreover, gender was a significant predictor of loneliness, with men reporting lower levels of loneliness compared to women. In contrast, age and race/ethnicity did not significantly predict loneliness, asocial beliefs, or mattering in this analysis.

**Table 2.** Path Coefficients.

Outcome	Predictor	*B*	*SE*	*z*	*p*	β	95% CI (LL, UL)
*Loneliness*	**Diagnostic group**	**8.726**	**1.482**	**5.886**	**<.001**	0.375	**[5.820, 11.631]**
	**Asocial beliefs**	**0.567**	**0.224**	**2.533**	.011	**0.165**	**[0.128, 1.006]**
	**Gender**	**−3.794**	**1.413**	−**2.685**	.007	**−0.163**	**[−6.564, −1.024]**
	Age	0.017	0.088	0.192	.848	0.012	[−0.156, 0.190]
	Race/ethnicity	−1.014	1.376	−0.737	.461	−0.043	[−3.710, 1.683]
*Asocial beliefs*	**Diagnostic group**	**3.114**	**0.588**	**5.298**	**<.001**	0.460	**[1.962, 4.266]**
	Gender	0.160	0.564	0.283	.777	0.024	[−0.945, 1.264]
	Age	−0.000	0.036	−0.012	.990	−0.001	[−0.072, 0.071]
	Race/ethnicity	−0.715	0.564	−1.268	.205	−0.104	[−1.821, 0.391]
*Mattering*	**Asocial beliefs**	**−0.482**	**0.091**	**−5.286**	**<.001**	**−0.409**	**[−0.660, −0.303]**
	Gender	−0.679	0.640	−1.062	.288	−0.085	[−1.933, 0.574]
	Age	−0.007	0.041	−0.162	.871	−0.013	[−0.087, 0.074]
	Race/ethnicity	0.117	0.641	0.182	.855	0.014	[−1.140, 1.373]
*Loneliness*	**Mattering**	**−1.340**	**0.218**	**−6.148**	**<.001**	**−0.460**	**[−1.767, −0.913]**

*Note*. For these analyses race/ethnicity was created as a dichotomous variable (0 = White [except non-white Latine], 1 = Non-White [i.e. Black/African-American, Hispanic/Latine, Asian, Indigenous American, Pacific Islander/Native Hawaiian, Bi-racial, and “Other”). Bolded path coefficients and test statistics represent a two-tailed significance of <.05.

## Discussion

The current study examined the pathways through which asocial beliefs and perceptions of mattering are associated with loneliness among people with schizophrenia and a non-SMI Comparison (NC) group. Consistent with our hypotheses, on average, the schizophrenia group reported higher levels of loneliness and asocial beliefs, as well as lower perceptions of mattering, relative to the NC group. Also consistent with our hypotheses, further analyses revealed that group differences in loneliness were partially accounted for by their associations with asocial beliefs and mattering. Specifically, people with schizophrenia not only experienced greater loneliness but were also more likely to report greater endorsement of asocial beliefs and to perceive less mattering by others, both of which were associated with increased loneliness. Importantly, these associations were not unique to schizophrenia; perceptions of mattering were associated with loneliness across both groups, suggesting it is a relevant correlate of loneliness in adults more broadly. However, the higher prevalence of asocial beliefs and lower mattering in the schizophrenia group indicates that these factors may play a particularly salient role in driving elevated loneliness in this population. These findings highlight modifiable psychological factors that could inform interventions aimed at reducing loneliness in schizophrenia.

Our findings are consistent with prior research indicating that people with schizophrenia experience elevated levels of loneliness (Badcock et al., 2015; Eglit et al., 2018; Michalska da Rocha et al., 2018). The strong negative association between the feeling that one matters to others and loneliness in both people with schizophrenia and the NC group aligns with previous studies in non-clinical populations, which have shown that individuals who feel valued and significant to others report lower loneliness (Flett et al., 2016; McComb et al., 2020). However, the current study extends prior work by demonstrating that this relationship also holds among people with schizophrenia, a group characterized by difficulties in social functioning. As perceptions of mattering may be modifiable (Bowman et al., 2022; Turner et al., 2024), future research should explore whether targeted interventions of enhancing perceptions of mattering can mitigate loneliness in people with schizophrenia.

In addition to lower mattering, people with schizophrenia reported greater endorsement of asocial beliefs. Importantly, asocial beliefs were associated with lower perceptions of mattering, suggesting a potential link between attitudes about social engagement and diminished feelings of being valued by others. It is worth noting that while asocial beliefs reflect either cognitive distortions (e.g. negative expectations about others) or motivational tendencies to avoid social interaction (e.g. the reduced drive for connection), these are conceptually distinct processes. Future research should clarify whether asocial beliefs reflect primarily cognitive distortions or motivational deficits, as distinguishing these processes may help guide the development of more targeted interventions to reduce loneliness. The measures employed in the current study do not allow us to disentangle these possibilities (e.g. whether higher asocial beliefs reflect a reduced motivation to seek social connection, or are shaped by secondary factors such as social anxiety or fear of rejection). The observed association between higher asocial beliefs and lower perceptions of mattering is consistent with prior research linking asocial beliefs to negative symptoms in schizophrenia, such as amotivation and asociality (Granholm et al., 2018; Grant & Beck, 2010).

Although not directly measured in the current study, our finding that asocial beliefs and lower perceptions of mattering partially mediated the association between schizophrenia and loneliness underscores the need to examine underlying cognitive mechanisms that may contribute to asocial beliefs and perceptions of mattering, particularly cognitive distortions related to social interactions. Cognitive distortions, such as misconceptions, misperceptions, or negative attributions about social interactions, may reinforce asocial beliefs and further discourage social participation. For example, people with schizophrenia may perceive themselves as unworthy of connection or misinterpret neutral social interactions as negative, reinforcing avoidance and deepening social isolation (Fulford & Holt, 2023). These cognitive distortions may, in turn, shape a preference for solitude or social withdrawal due to fear of rejection or social inadequacy, reinforcing a cycle of loneliness and social isolation (Cavieres et al., 2023; Fulford & Holt, 2023). These processes are consistent with theoretical models suggesting that negative social cognitions contribute to a self-reinforcing cycle of withdrawal and loneliness (Cacioppo et al., 2006; Hawkley & Cacioppo, 2010). From a cognitive-behavioral perspective, distorted beliefs about the self and others can drive avoidance and emotional distress (Beck, 1979). Importantly, avoidance of social interactions also reduces opportunities for social acknowledgement and affirmation, processes central to perceptions of mattering (Flett, 2022; Taylor & Turner, 2001). For example, according to the predominant model of loneliness from Cacioppo and colleagues, negative expectations about social interactions contribute to avoidance and withdrawal, and can thereby intensify loneliness (Cacioppo & Patrick, 2008; Cacioppo et al., 2006; Hawkley & Cacioppo, 2010). Within this context, these frameworks may suggest that asocial beliefs may diminish one’s sense of mattering to others, which in turn exacerbates feelings of loneliness. Future research should explore whether interventions targeting cognitive distortions could help disrupt this cycle and improve social outcomes for people with schizophrenia.

The indirect effect of diagnostic group on loneliness through asocial beliefs and mattering was modest ( β = .086), suggesting that additional factors such as social motivation deficits or stigma-related experiences could contribute to loneliness in schizophrenia beyond those included in the current analyses. While social motivation deficits are known to contribute to social withdrawal in schizophrenia (Catalano & Green, 2023; Gentry & Palmer, 2022), further research is needed to determine their specific pathways in the relationship between mattering and loneliness. Future research should examine how positive and negative social motivation, social cognition, cognitive distortions, and social attribution biases contribute to asocial beliefs and perceptions of mattering. This may help further clarify intervention targets for reducing loneliness in schizophrenia.

### Limitations

These findings should be interpreted in light of several limitations. First, due to the cross-sectional design, the results cannot establish causal relationships between asocial beliefs, mattering, and loneliness. Future longitudinal studies, particularly those incorporating interventions or naturalistic changes in these constructs, are needed to clarify temporal needed to clarify temporal relationships, causal mechanisms, and modifiability. Second, model fit was mixed. While some indices suggested acceptable fit (i.e. χ², CFI, and SRMR), the RMSEA (0.126), and TLI (0.784) exceeded conventional thresholds for goodness of fit, indicating that the model may not fully capture the underlying data structure and should be interpreted with caution. These fit indices could reflect model limitations or constraints imposed by the relatively small sample size, particularly given the number of estimated parameters. Larger samples in future studies may be needed to refine the model and improve model fit. Finally, the sample composition limited the ability to examine potential moderators of these associations, including age, gender, and race/ethnicity. Although race/ethnicity was not a significant predictor in the present study, the greater diversity in the schizophrenia group underscores the need for research on the intersection of loneliness, social connection, cultural norms, stigma, and SMI. Given that ethno-racial and socio-economic disparities impact access to mental health care and social support (Holt-Lunstad et al., 2017; Levine, 2018; Murthy, 2017), future research should explore how these factors shape loneliness in schizophrenia. Similarly, gender-related differences in social functioning, onset, symptom presentation, and loneliness warrant further exploration. Although some studies suggest minimal gender differences in indirect measures of loneliness (Lee et al., 2019), direct self-reported loneliness may be more frequently endorsed by women (Lee et al., 2019; Reinwarth et al., 2023). Investigating whether gender moderates these relationships among asocial beliefs, mattering, and loneliness may inform the development of tailored interventions for people with schizophrenia.

In sum, despite the above interpretive caveats, the current study provides some novel insights into mechanisms underlying loneliness in schizophrenia, highlighting the roles of asocial beliefs and perceptions of mattering. These findings complement prior research on elevated loneliness in schizophrenia by identifying potentially modifiable psychological factors that contribute to this experience. Traditional interventions often focus on enhancing social skills and increasing social contact; they may not fully address the subjective experience of loneliness, which is influenced by perceptions of social value, belonging, and connection rather than solely the quantity of social interactions (Gentry & Palmer, 2021). Even with increased social engagement, individuals may continue to feel lonely if they do not perceive themselves as meaningful or valued members of their social environment. Therefore, interventions that go beyond social contact and instead focus on enhancing perceptions of social value and maladaptive beliefs may be particularly effective. Our findings may highlight the potential effectiveness of interventions targeting both perceptions of mattering and maladaptive social cognitions (i.e. asocial beliefs). Cognitive-behavioral interventions, for example, have been shown to reduce loneliness in part by addressing distorted beliefs about social interactions (Käll & Andersson, 2023; Masi et al., 2011).

Moreover, experiences of stigma, social rejection, and internalized negative beliefs may hinder perceptions of mattering, necessitating targeted strategies to address these barriers in schizophrenia and other forms of SMI (Bell et al., 2020; Cavieres et al., 2023; Michalska da Rocha et al., 2018). Future research should examine whether mattering-focused interventions can be adapted for individuals with schizophrenia and whether integrating strategies that address asocial beliefs alongside mattering-based approaches yields stronger outcomes. By identifying psychological factors that contribute to loneliness, the current study lays the groundwork for more effective, targeted interventions that promote social well-being and mitigate the detrimental effects of loneliness in schizophrenia.
